# Fox Insight: Most Bothersome Symptoms in Early‐Stage Parkinson's Disease

**DOI:** 10.1002/mdc3.14321

**Published:** 2025-01-03

**Authors:** Aaron Lerner, Jennifer R. Mammen, Mirinda Tyo, Peggy Auinger, Raunak Al‐Rubayie, Yuge Xiao, Connie Marras, Jamie L. Adams

**Affiliations:** ^1^ University of Rochester Medical Center, Center for Health + Technology (CHeT) Rochester New York USA; ^2^ College of Nursing and Health Sciences University of Massachusetts Dartmouth North Dartmouth Massachusetts USA; ^3^ Department of Neurology University of Rochester Medical Center Rochester New York USA; ^4^ The Michael J. Fox Foundation for Parkinson's Research New York New York USA; ^5^ The Edmond J Safra Program in Parkinson's Disease and Morton and Gloria Shulman Movement Disorders Clinic Toronto ON Canada

**Keywords:** early Parkinson's, symptoms

## Abstract

**Background:**

Limited evidence exists regarding the meaningfulness of symptoms experienced in early Parkinson's disease (PD).

**Objectives:**

To identify the most bothersome symptoms experienced by people with early PD, leveraging data from the Parkinson's Disease Patient Report of Problems (PD‐PROP) questionnaire within the Fox Insight Study.

**Methods:**

Individuals with a self‐reported diagnosis of PD completed the PD‐PROP questionnaire, reporting up to five most bothersome symptoms. Symptom types and frequencies were derived through a combination of human expertise and machine learning.

**Results:**

Participants (*N* = 8536) were 0.9 years since diagnosis, predominantly white (96%), male (53.3%), and with an average age of 64.6 years. Top most bothersome motor symptoms were tremor (55.9%) and gait issues (36.7%). Top most bothersome non‐motor symptoms were pain/discomfort (33.1%) and physical fatigue (27.5%).

**Conclusions:**

This study underscores the complexity of early PD symptomatology. Future consideration of diverse patient experiences is needed to improve therapeutic and outcome measurement strategies.

People with Parkinson's disease (PD) experience a broad range of symptoms, from cardinal motor symptoms (ie, tremor, stiffness, bradykinesia, postural instability) to non‐motor symptoms that affect speech, cognition, and sleep, among others.^1^ Evidence indicates that symptom patterns are highly individual, change with disease duration and progression, and may be more or less bothersome at different stages.^2^, ^3^ This variability has presented challenges in determining which symptoms are most meaningful and most appropriate to monitor for research and clinical practice.

With emphasis on earlier detection and monitoring, it is imperative to understand meaningful symptoms of early disease (≤2 years since diagnosis), as distinct from later stages.^4^ Recent studies have focused on symptom prevalence in early Parkinson's disease (PD); however, limited evidence exists regarding the importance of these experiences, which is essential for an overall assessment of meaningfulness—necessary for the development of relevant outcome measures and therapeutic treatments for PD.^5^, ^6^, ^7^, ^8^ Patient‐reported outcomes (PROs) can support this type of evaluation, but most have limited flexibility and may not adequately reflect symptom experiences.^9^, ^10^ By contrast, use of flexible, open‐ended PROs offers the ability to capture a more individualized range of patient data and could be more responsive to and reflective of the lived experience. However, availability and use of open‐ended PROs remains scarce, and the complexity of managing and analyzing data from these tools has presented significant challenges.^11^ The Parkinson's Disease Patient Report of Problems (PD‐PROP) questionnaire is an open‐ended PRO designed to identify the most bothersome issues faced by patients. This tool has the ability to collect data on what bothers people most, without the bias inherent in fixed‐response PROs that assess pre‐specified symptoms.^9^ Thus, the purpose of this study was to identify which symptoms of PD are most bothersome to people with early disease using PD‐PROP data collected during the Fox Insight Study.

## Methods

This study is an analysis of Fox Insight survey data. The Fox Insight platform is an ongoing digital observational study gathering anonymized survey data on health, lifestyle, and past exposures from people with and without self‐reported PD.^12^ As part of this study, participants with PD completed demographic questionnaires and the PD‐PROP 2.0.^13^ Informed electronic consent was obtained with IRB approval from New England/WCG Institutional Review Board (IRB#:120160179).

### Participant Selection and Eligibility Criteria

Adults aged >18 years, with and without a self‐reported diagnosis of PD, were eligible for the Fox Insight study, which began data collection in 2017. This study relied on self‐reported clinical diagnosis of PD, which has been previously shown to correlate with clinician‐determined PD.^14^ The recruitment process included social media campaigns, referrals from clinicians, and other methods.^13^ The PD‐PROP instrument is administered to participants every 3 months. Only data from participant's first completed PD‐PROP 2.0, within 2 years of PD diagnosis, were included in the present analysis. Data were retrieved May 2023.

### PD‐PROP Platform and Curation

PD‐PROP is an online survey platform that asks people with PD to describe, in their own words, up to five most bothersome problems of PD and how these affect their daily lives. Questions were open ended and response length was unlimited: (1) “What is the most bothersome problem for you due to your Parkinson's disease?” and (2) “In what way does this problem bother you by affecting your everyday functioning or ability to accomplish what needs to be done?”

For curation, de‐identified responses were analyzed using techniques created by PD‐PROP developers for symptom classification, which combines human expertise with natural language processing and machine learning.^9^, ^15^ Findings from Marras et al (2023) support the robustness and accuracy of the developed algorithm.^9^

### Data Analysis

Descriptive statistics were computed for demographics and symptoms, with symptoms frequencies ranked from most to least bothersome (1st to 5th) as indicated by the respondent. Symptoms identified as part of any of the five bothersome problems were considered as actively bothersome to any degree at the current PD stage. Chi‐square tests were used to compare proportions of the most frequently reported bothersome motor and non‐motor concerns by demographic characteristics and overall concerns stratified by age categories, sex, and race are reported.

## Results

A total of 8536 participants with PD ≤2 years since diagnosis were included in the analysis (mean duration 0.9 years; SD = 0.6). Participants were predominantly White/Caucasian (96.0%), with an average age of 64.6 years (SD = 10.2) and a slight male majority (53.3%). Demographic characteristics are shown in Table 1.

**TABLE 1:** Demographics of Fox Insight participants early PD ≤2 years from diagnosis

	N* = 8536*
Age, year mean (SD)	64.6 (10.2)
Years since diagnosis, mean (SD)	0.9 (0.6)
Male, n (%)	4553 (53.3)
Race, n (%)	
White or Caucasian	8197 (96.0)
Black or African American	45 (0.5)
American Indian or Alaska Native	33 (0.4)
Asian	107 (1.3)
Native Hawaiian or Other Pacific Islander	6 (0.1)
Multiple race	90 (1.1)
Prefer not to answer	58 (0.7)
Hispanic ethnicity, n (%)	460 (5.4)

**Figure 1:**
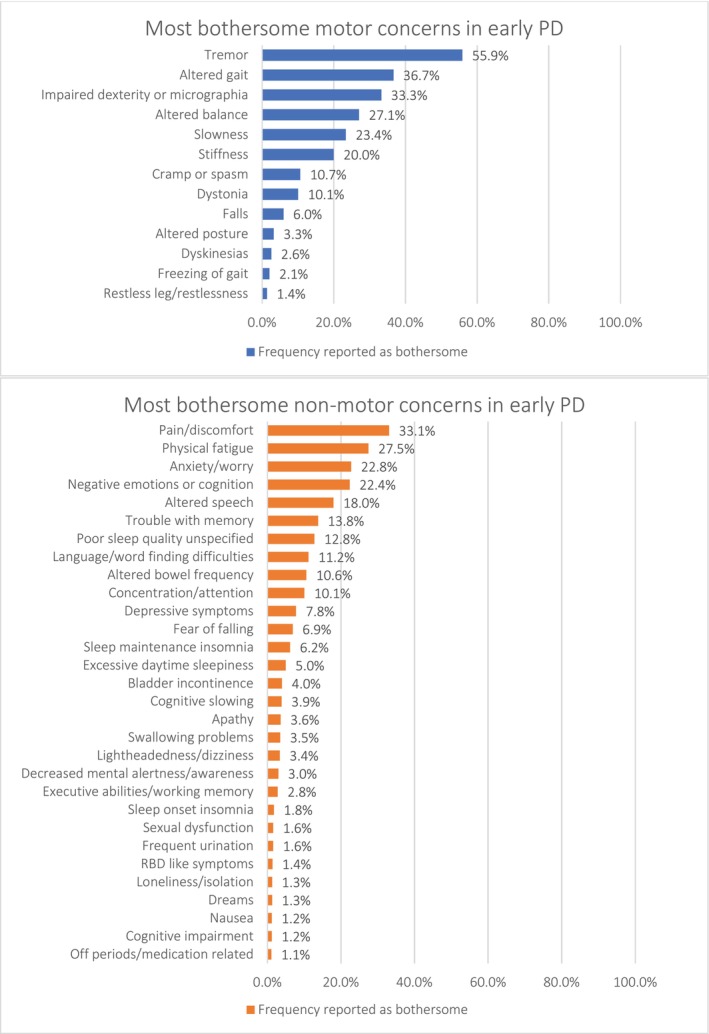
Most bothersome motor and non‐motor concerns in early PD ≤2 years from diagnosis. The following symptoms were cited as bothersome by <1% of people with early PD, less than 2 years from diagnosis: Altered facial expression (0.9%), Headache (0.8%), Mental fatigue (0.8%), Bloating/feeling full (0.8%), Off periods/medication not mentioned (0.7%), Excessive sweating (0.7%).

As shown in Fig. 1, the most often reported bothersome motor concern was tremor (55.9%). This was followed by altered gait (36.7%), impaired dexterity or micrographia (33.3%), balance (27.1%), slowness (23.4%) and stiffness (20.0%). The most reported non‐motor symptoms were pain/discomfort (33.1%), physical fatigue (27.5%), anxiety/worry (22.8%), and negative emotions or cognition (22.4%). Other symptoms, such as cramp or spasm, dystonia, altered speech, and trouble with memory were less frequently cited as bothersome.

A significant difference was found based on both age and sex for top symptoms of tremor and pain. In general, younger participants (20–49 years) reported highest percentages of tremor (62.1%) and pain/discomfort (47.3%), with declining reports of tremor and pain observed in older age groups (*P* < 0.001), as shown in Table S1. In participants 70–79 and 80+ years, physical fatigue (27% and 26.9%, respectively) is the most reported non‐motor symptom, rather than pain/discomfort. In participants 80+ years, altered balance (45.7%) is the most frequently reported motor symptom, rather than tremor. Compared to males, females reported higher frequency of pain/discomfort (39.8% vs. 27.2%, respectively; *P* < 0.01) with similar findings for tremor (57.5% vs. 54.6%; *P* < 0.01). Although the difference in tremor frequency by sex is statistically significant, we believe it lacks clinical/practical significance. Detailed frequencies of symptoms by age groups are shown in Supplementary Figure S1–S5 with frequencies of symptoms by sex shown in Figures S6 and S7. There were no significant differences in the reporting of tremor or pain by race/ethnicity. This may be due to a lack of power, as frequencies by race/ethnicity may suggest potential differences. Frequencies of symptoms stratified by race/ethnicity are shown in Figure S8–S13.

## Discussion

This brief report of most bothersome symptoms in early PD represents the largest cohort with qualitative data to date. Existing information in this area has been primarily obtained from smaller studies, with limited evidence to show what is meaningful from the patient perspective.^5^, ^6^, ^8^ Prior research indicates bothersomeness is highly subjective and might depend on the symptom's impact on quality of life and daily activities.^5^ Large sample studies of patient experiences, such as presented here, can improve understanding of what matters at different stages of disease, guide development of relevant clinical measures, and support regulatory decision‐making.^16^, ^17^

In our study, motor and non‐motor symptoms were both widespread and bothersome in early PD. Tremor was most cited overall, which agrees with findings from other studies.^3^, ^5^, ^6^, ^8^, ^9^ However, gait changes and balance issues were also common, which have historically been more often attributed to individuals >2 years from diagnosis.^3^ Consistent with other studies, we also observed substantial non‐motor concern (eg, pain/discomfort, physical fatigue, and anxiety/worry), which impact quality of life and daily activities.^5^, ^18^ Our findings, along with growing evidence from the literature, suggest a need to prioritize the assessment of non‐motor symptoms from the earliest stages and onwards.^3^, ^6^, ^18^

In this study, we found significant variations in top concerns based on age and sex. This suggests that PD affects individuals differently as they age, with notable differences observed within the earliest stages of the disease. Decreasing reports of tremor and pain/discomfort with age might reflect normalization, changes in physical health, psychological adaptation to the disease, or a focus on other diseases/co‐morbidities.^19^ Observed sex‐specific differences in bothersome symptoms also underscore the need for personalized treatment approaches to best address the experiences of each patient. Cumulatively, these findings demonstrate the importance of considering demographics in PD research and care.

This study also highlights the value of utilizing open‐response PROs to understand the priorities of individuals affected by early PD, particularly when existing instruments may not adequately capture their experiences.^20^ Work by Miller et al (2020) in multiple sclerosis suggests that fixed‐response PROs overlook important elements, such as social challenges and disease management issues, emphasizing the limitations of restricting participant responses to pre‐specified concepts.^10^ The novel open‐ended approach used here allows for more flexible and humanistic interpretation than is possible with fixed PROs, in a low burden, cost‐effective, and scalable format—making this a valuable resource for collection of longitudinal qualitative data.^3^, ^9^, ^15^, ^21^, ^22^ Use in clinical settings may also be feasible and has the potential to contribute to patient‐centric care and clinical goal setting.^15^

### Limitations

This study was conducted in a largely White/Caucasian sample and might not represent a more diverse PD population. The use of an online text‐based platform for collection of PD patient‐reported outcomes could also deter individuals with dexterity challenges. Furthermore, machine curation is dependent on algorithms built using existing knowledge and curator subjectivity.^9^ Thus, normative bias may also inadvertently prioritize symptoms that are better recognized, potentially overlooking less common symptoms of early PD.^9^ In light of recent qualitative work, certain grouper domains such as “Other Motor,” may lack specificity and obscure understanding of conceptually distinct symptoms and impacts.^3^, ^5^, ^6^, ^8^ Considering the self‐reported diagnosis nature of PD‐PROP and the observed frequency of gait abnormalities, there is potential that participants may be exhibiting early‐stage atypical Parkinsonism rather than PD. Differentiating between these conditions remains a persistent challenge that necessitates further investigation, as accurate diagnosis typically requires additional imaging and physical examination.^23^ Future studies focusing on gait impairment in early‐stage disease and comparing PROs with physician‐based measures may provide clearer differentiation. Despite these limitations, we believe the use of open‐ended flexible PROs offers a valuable opportunity to effectively and efficiently explore symptom experiences and contribute to growing knowledge of what matters to individuals with PD.

People with PD experience a range of motor and non‐motor symptoms that are actively bothersome in early disease, in particular tremor, gait changes, fine motor issues, and pain, which may vary by age and biological sex. Use of a flexible, open‐response PRO, such as PD‐PROP, which captures verbatim patient experiences, can contribute to understanding what matters to people with PD on a larger scale than has been previously possible. This knowledge can help to guide identification of meaningful symptoms and inform development of future outcomes assessments, clinical monitoring, and health care delivery. Iterative refinement in curation will likely be needed to achieve more precise understanding of meaningful symptom patterns over time.

## Author Roles

(1) Research project: A. Conception, B. Organization, C. Execution; (2) Statistical Analysis: A. Design, B. Execution, C. Review and Critique; (3) Manuscript Preparation: A. Writing of the first draft, B. Review and Critique.

A.L.: 1C, 2A, 2C, 3A, 3B.

J.R.F.: 1A, 1B, 1C, 2A, 2C, 3B.

M.T.: 3B.

P.A.: 2A, 2B, 2C, 3B.

R.A.: 3B.

Y.X.: 1A, 1B, 1C, 3B.

C.M.: 2A, 2C, 3B.

J.L.: 1A, 1B, 1C, 2A, 2C, 3B.

## Disclosures

**Ethical Compliance Statement:** Institutional review board—Institutional review board approval was obtained by the New England/WCG Institutional Review Board (IRB#:120160179). Declaration of Patient Consent—Informed electronic consent was obtained via the Fox Insight website. Affirmation of Journal's Ethical Publication Guidelines—We confirm that we have read the Journal's position on issues involved in ethical publication and affirm that this work is consistent with those guidelines.

**Funding Sources and Conflicts of Interest:** This study was funded by the Michael J. Fox Foundation. The contents are those of the author(s) and do not necessarily represent the official views of, nor an endorsement by, FDA/HHS or the U.S. Government. The authors declare that there are no conflicts of interest relevant to this work.

**Financial Disclosures for the Previous 12 Months:** The authors declare that there are no additional disclosures to report.

## Supporting information


**TABLE S1.** Most bothersome motor and non‐motor concerns by demographic characteristics.
**Figure S1.** Age 20–49 years: Most bothersome motor and non‐motor concerns in early PD ≤2 years from diagnosis (n = 778). Symptoms cited as bothersome by <1% of people with early PD, less than 2 years from diagnosis are not shown.
**Figure S2.** Age 50–59 years: Most bothersome motor and non‐motor concerns in early PD ≤2 years from diagnosis (n = 1685). Symptoms cited as bothersome by <1% of people with early PD, less than 2 years from diagnosis are not shown.
**Figure S3.** Age 60–69 years: Most bothersome motor and non‐motor concerns in early PD ≤2 years from diagnosis (n = 3319). Symptoms cited as bothersome by <1% of people with early PD, less than 2 years from diagnosis are not shown.
**Figure S4.** Age 70–79 years: Most bothersome motor and non‐motor concerns in early PD ≤2 years from diagnosis (n = 2371). Symptoms cited as bothersome by <1% of people with early PD, less than 2 years from diagnosis are not shown.
**Figure S5.** Age 80+ years: Most bothersome motor and non‐motor concerns in early PD ≤2 years from diagnosis (n = 383). Symptoms cited as bothersome by <1% of people with early PD, less than 2 years from diagnosis are not shown.
**Figure S6.** Males: Most bothersome motor and non‐motor concerns in early PD ≤2 years from diagnosis (n = 4553). Symptoms cited as bothersome by <1% of people with early PD, less than 2 years from diagnosis are not shown.
**Figure S7.** Females: Most bothersome motor and non‐motor concerns in early PD ≤2 years from diagnosis (n = 3983). Symptoms cited as bothersome by <1% of people with early PD, less than 2 years from diagnosis are not shown.
**Figure S8.** White or Caucasian: Most bothersome motor and non‐motor concerns in early PD ≤2 years from diagnosis (n = 8197). Symptoms cited as bothersome by <1% of people with early PD, less than 2 years from diagnosis are not shown.
**Figure S9.** Black or African American: Most bothersome motor and non‐motor concerns in early PD ≤2 years from diagnosis (n = 45). Symptoms cited as bothersome by <1% of people with early PD, less than 2 years from diagnosis are not shown.
**Figure S10.** American Indian or Alaska Native: Most bothersome motor and non‐motor concerns in early PD ≤2 years from diagnosis (n = 33). Symptoms cited as bothersome by <1% of people with early PD, less than 2 years from diagnosis are not shown.
**Figure S11.** Asian: Most bothersome motor and non‐motor concerns in early PD ≤2 years from diagnosis (n = 107). Symptoms cited as bothersome by <1% of people with early PD, less than 2 years from diagnosis are not shown.
**Figure S12.** Native Hawaiian or Other Pacific Islander: Most bothersome motor and non‐motor concerns in early PD ≤2 years from diagnosis (n = 6). Symptoms cited as bothersome by <1% of people with early PD, less than 2 years from diagnosis are not shown.
**Figure S13.** Multiple Race: Most bothersome motor and non‐motor concerns in early PD ≤2 years from diagnosis (n = 90). Symptoms cited as bothersome by <1% of people with early PD, less than 2 years from diagnosis are not shown.

## Data Availability

The data that support the findings of this study are openly available in Fox Insight Date Exploration Network at http://doi.org/10.17616/R31NJML7, reference number SCR_022321.
