# Supplementary material for: Fox Insight: Most Bothersome Symptoms in Early‐Stage Parkinson's Disease
Source: Mov Disord Clin Pract. 2025 Jan 3;12(4):510–5. doi: 10.1002/mdc3.14321 (PMC11998684; doi:10.1002/mdc3.14321)
Supplement: Supplementary file 1 — TABLE S1. Most bothersome motor and non‐motor concerns by demographic characteristics. Figure S1. Age 20–49 years: Most bothersome motor and non‐motor concerns in early PD ≤2 years from diagnosis (n = 778). Symptoms cited as bothersome by <1% of people with early PD, less than 2 years from diagnosis are not shown. Figure S2. Age 50–59 years: Most bothersome motor and non‐motor concerns in early PD ≤2 years from diagnosis (n = 1685). Symptoms cited as bothersome by <1% of people with early PD, less than 2 years from diagnosis are not shown. Figure S3. Age 60–69 years: Most bothersome motor and non‐motor concerns in early PD ≤2 years from diagnosis (n = 3319). Symptoms cited as bothersome by <1% of people with early PD, less than 2 years from diagnosis are not shown. Figure S4. Age 70–79 years: Most bothersome motor and non‐motor concerns in early PD ≤2 years from diagnosis (n = 2371). Symptoms cited as bothersome by <1% of people with early PD, less than 2 years from diagnosis are not shown. Figure S5. Age 80+ years: Most bothersome motor and non‐motor concerns in early PD ≤2 years from diagnosis (n = 383). Symptoms cited as bothersome by <1% of people with early PD, less than 2 years from diagnosis are not shown. Figure S6. Males: Most bothersome motor and non‐motor concerns in early PD ≤2 years from diagnosis (n = 4553). Symptoms cited as bothersome by <1% of people with early PD, less than 2 years from diagnosis are not shown. Figure S7. Females: Most bothersome motor and non‐motor concerns in early PD ≤2 years from diagnosis (n = 3983). Symptoms cited as bothersome by <1% of people with early PD, less than 2 years from diagnosis are not shown. Figure S8. White or Caucasian: Most bothersome motor and non‐motor concerns in early PD ≤2 years from diagnosis (n = 8197). Symptoms cited as bothersome by <1% of people with early PD, less than 2 years from diagnosis are not shown. Figure S9. Black or African American: Most bothersome motor and non‐motor concerns in early [file MDC3-12-510-s001.docx]

**Supplementary Table 1.** Most frequent overall bothersome motor and non-motor concerns in early PD <2 years from diagnosis by demographic characteristics

|  | n | Motor  % Tremor | Non-motor  % Pain/discomfort |
| --- | --- | --- | --- |
| Overall | 8536 | 55.9 | 33.1 |
| Age, y |  | **p<0.001** | **p<0.001** |
| 20-49 | 778 | 62.1 | 47.3 |
| 50-59 | 1685 | 59.8 | 41.2 |
| 60-69 | 3319 | 58.8 | 33.6 |
| 70-79 | 2371 | 49.7 | 23.9 |
| 80+ | 383 | 39.7 | 20.6 |
| Biological Sex |  | **p=0.01** | **p<0.001** |
| Male | 4553 | 54.6 | 27.2 |
| Female | 3983 | 57.5 | 39.8 |
| Race |  | p=0.67 | p=0.25 |
| White or Caucasian | 8197 | 55.9 | 33.1 |
| Black or African American | 45 | 51.1 | 35.6 |
| American Indian or Alaska Native | 33 | 69.7 | 21.2 |
| Asian | 107 | 54.2 | 29.0 |
| Native Hawaiian or Other Pacific Islander | 6 | 50.0 | 66.7 |
| Multiple race | 90 | 55.6 | 36.7 |
| Ethnicity |  | p=0.42 | p=0.67 |
| Hispanic | 460 | 54.1 | 32.2 |
| Non-Hispanic | 8076 | 56.0 | 33.1 |

**Supplementary Figure 1. Age 20-49 years**: Most bothersome motor and non-motor concerns in early PD <2 years from diagnosis (n=778)

**Supplementary Figure 2. Age 50-59 years**: Most bothersome motor and non-motor concerns in early PD <2 years from diagnosis (n=1,685)

**Supplementary Figure 3. Age 60-69 years**: Most bothersome motor and non-motor concerns in early PD <2 years from diagnosis (n=3,319)

**Supplementary Figure 4. Age 70-79 years**: Most bothersome motor and non-motor concerns in early PD <2 years from diagnosis (n=2,371)

**Supplementary Figure 5. Age 80+ years**: Most bothersome motor and non-motor concerns in early PD <2 years from diagnosis (n=383)

**Supplementary Figure 6. Males**: Most bothersome motor and non-motor concerns in early PD <2 years from diagnosis (n=4,553)

**Supplementary Figure 7. Females**: Most bothersome motor and non-motor concerns in early PD <2 years from diagnosis (n=3,983)

**Supplementary Figure 8. White or Caucasian**: Most bothersome motor and non-motor concerns in early PD <2 years from diagnosis (n=8,197)

**Supplementary Figure 9. Black or African American**: Most bothersome motor and non-motor concerns in early PD <2 years from diagnosis (n=45)

**Supplementary Figure 10. American Indian or Alaska Native**: Most bothersome motor and non-motor concerns in early PD <2 years from diagnosis (n=33)

**Supplementary Figure 11. Asian**: Most bothersome motor and non-motor concerns in early PD <2 years from diagnosis (n=107)

**Supplementary Figure 12. Native Hawaiian or Other Pacific Islander**: Most bothersome motor and non-motor concerns in early PD <2 years from diagnosis (n=6)

**Supplementary Figure 13. Multiple Race**: Most bothersome motor and non-motor concerns in early PD <2 years from diagnosis (n=90)
